# Estimating the prevalence of chronic kidney disease in the older population using health screening data in Japan

**DOI:** 10.1007/s10157-024-02570-y

**Published:** 2024-10-05

**Authors:** Arisa Kobayashi, Keita Hirano, Tadahisa Okuda, Tatsuyoshi Ikenoue, Takashi Yokoo, Shingo Fukuma

**Affiliations:** 1 Human Health Sciences, Kyoto University Graduate School of Medicine, 53 Shogoin Kawahara-Cho, Sakyo-Ku, Kyoto-Shi, Kyoto, 606-8057 Japan; 2 Division of Nephrology and Hypertension, Department of Internal Medicine, The Jikei University School of Medicine, Tokyo, Japan; 3 Department of Health Data Science, Tokyo Medical University, Tokyo, Japan; 4 Data Science and AI Innovation Research Promotion Center, Shiga University, Hikone, Japan; 5 Department of Epidemiology Infectious Disease Control and Prevention, Hiroshima University Graduate School of Biomedical and Health Sciences, Hiroshima, Japan

**Keywords:** Chronic kidney disease, Prevalence, Older population, Selection bias, Inverse probability weighting

## Abstract

**Background:**

In aging societies, the prevalence of chronic kidney disease (CKD) is expected to increase but may be underestimated because many asymptomatic patients remain undiagnosed. This study aimed to estimate the CKD prevalence among the general older population in Japan.

**Methods:**

This cross-sectional study used health screening data from the Japan Health Insurance Association collected between April 2014 and March 2023. Data from older people aged 65–90 years who underwent renal function screening for estimated glomerular filtration rate (eGFR) and urine protein tests were analyzed. CKD was defined as eGFR < 60 mL/min/1.73 m^2^ or proteinuria ≥ 1 + . Inverse probability weighting was used to account for the selection bias. The variables used for weighting were age, sex, insurance status, and the number of previous screenings.

**Results:**

Among 2.98 million older individuals, 588,809 (19.7%) had undergone screening (median [IQR] age, 69.9 [67.9–76.2] years, 337,862 women [57.4%]). Regarding the weighted CKD prevalence, 25.3% of the individuals aged 65–90 years had CKD; 11.8% of those aged 65–75 years and 34.6% of those aged 75 years and over showed an increase in prevalence with age. Among the patients with CKD, over half exhibited mild renal dysfunction without proteinuria. Hypertension and diabetes were common comorbidities in older patients with CKD.

**Conclusions:**

This cross-sectional study revealed that the weighted prevalence of CKD in the older population aged 65–90 years was high (one in four individuals), indicating that it increases with age. Further studies are required to examine the clinical significance of these findings.

**Supplementary Information:**

The online version contains supplementary material available at 10.1007/s10157-024-02570-y.

## Introduction

Chronic kidney disease (CKD) is a substantial global health concern and a major public health challenge worldwide today. CKD is often undiagnosed and untreated until it reaches an advanced stage because it usually has no symptoms [1]. Also, the number of patients undergoing chronic dialysis in Japan continues to increase as more people with CKD progress to stages requiring renal replacement therapy. In addition, the average age of both new and chronic dialysis patients is getting older as the population ages [2]. Patients with CKD are at a high risk of cardiovascular diseases [3–6], and understanding the prevalence of CKD is critical because of its impact on various conditions such as reduced quality of life and excessive utilization of healthcare resources, in addition to its critical effect on mortality.

In a previous study reported 15 years ago, the prevalence of CKD in Japan was approximately 13% [7]. While this estimate has been widely used to discuss the high prevalence of CKD, it is over a decade old and may differ from recent trends, indicating that the prevalence of CKD is rising because of various factors, such as increased life expectancy and a higher incidence of hypertension, diabetes, and other lifestyle-related diseases [8–10]. CKD is common in the older population because of the physiological decline in renal function and accumulation of comorbidities [11], yet it has received inadequate medical attention. Thus, the actual prevalence of CKD may have been substantially underestimated, particularly in older populations. The aging of the Japanese population could significantly affect the prevalence of CKD, and the incidence of age-related diseases, including CKD, is expected to increase. Understanding the actual status of these diseases is necessary; however, the status and prevalence of CKD in the older population remain unclear.

This study aimed to fill this knowledge gap by providing updated estimates of the prevalence of CKD among the older population in Japan using health screening data. However, when using health screening data, the estimate is strongly influenced by selection bias due to the more health-conscious nature of the group, potentially leading to an underestimation of CKD prevalence. To address this issue, we employed the inverse probability weighting (IPW) method. By using this statistical method to account for selection bias, this study provides a clearer understanding of CKD prevalence and enhances our knowledge of its epidemiology in an aging society.

## Materials and methods

### Data source and setting

This study aimed to determine the prevalence of CKD in the older population in Japan. Data from the national health examination cohort between April 2014 and March 2023 provided by DeSC Healthcare, Inc. (Tokyo, Japan), were used for the analysis. The database includes health insurance claims data from three types of insurers: (1) national health insurance for non-employees and individual proprietors; (2) health insurance for employees of large corporations; and (3) the Advanced Elderly Medical Service System for those aged 75 and older. It contains information on approximately 12,000,000 individuals distributed throughout Japan, encompassing a wide range of age groups, including young, middle-aged, and elderly individuals. Therefore, the age distribution in the DeSC database closely aligns with the estimates for the Japanese population [12]. The data included demographic characteristics such as age, sex, and body mass index (BMI); clinical information, such as systolic blood pressure, diastolic blood pressure, hemoglobin A1c (HbA1c), low-density lipoprotein cholesterol (LDL-C), creatinine, and urinalysis data; and self-administered medical questionnaire items such as whether the patient takes medication for hypertension, diabetes, or dyslipidemia, and current smoking status. eGFR values were calculated using the Chronic Kidney Disease Epidemiology Collaboration equation for Japanese individuals [13]. CKD was defined as eGFR less than 60 mL/min/1.73 m^2^ or proteinuria 1 + or greater by a dip-stick test. All information was anonymized at the time it was provided, and could be followed up anonymously if there were no changes in health insurance. This study was conducted in accordance with the STROBE guidelines. Since we analyzed only anonymized data, the need for ethics committee approval and informed consent was waived.

### Participant selection

We included individuals aged between 65 and 90 years old. The baseline period was April 2017–March 2018, and follow-up was conducted until March 2023. The health screening group was defined as individuals who had undergone health screenings after the baseline period, had at least three years of insurance registration prior to the date of the first health screening, and had at least two measurements of creatinine and urinary protein levels (Online resource Fig. 1). People with missing basic clinical information were excluded.

### Estimation of prevalence

To account for the effect of the selection bias that those who underwent health screening are a biased population, we used the IPW method. We estimated the probability of not undergoing health screening (probability of missing health screening data) based on the available characteristics, and the inverse probability was used to estimate the prevalence in the entire population. To assess the probability, we used a logistic regression model. We adopted age, sex, whether the person is the insured or a dependent, and the number of previous health screenings as explanatory variables. The number of previous health screenings was defined as the number of screenings performed before the baseline period. If the first health screening after the baseline period was not included in this period, the number of previous health screenings was defined as the number of health screenings conducted three years prior to the first post-baseline health screening.

### Statistical analysis

Patient characteristics are presented as mean ± standard deviation or median (interquartile range) for continuous variables and numerical values (percentages) for categorical variables. In this study, a calibration analysis was performed to assess the validity of the forecasting model. Calibration plots were created to test the agreement between predicted probabilities and actual outcomes. For the analysis, the data were divided into adequate rank groups and the mean predicted probabilities were compared to the proportion of observed probabilities in each group. This process allowed for a visual assessment of the match between the predictions of the model and observed data. All statistical analysis were performed using STATA MP (version 18.0; STATA Corporation, College Station, TX, US). For all analyses, a two-tailed p value < 0.05 was considered statistically significant.

## Results

### Study population

In total, 2.98 million participants were included in this study, and 588,809 people underwent health screening (Table 1). Overall, the median age was 69.9 (interquartile range (IQR), 67.9–76.2), and females accounted for 57.4% of the participants. A total of 334,240 participants (56.8%) had hypertension, 62,351 (14.7%) had diabetes, and 283,555 (48.4%) had dyslipidemia.

**Table 1:** Characteristics of participants

	Total *n* = 2,981,750	Health screening group *n* = 588,809
Age [years (IQR)]	76.2 (69.6–81.1)	69.9 (67.9–76.2)
Female [n (%)]	1,709,560 (57.3)	337,862 (57.4)
HT [n (%)]	–	334,240 (56.8)
DM [n (%)]	–	62,351 (14.7)
HL [n (%)]	–	283,555 (48.4)

*HT* Hypertension, *DM* Diabetes mellitus, *HL* Hyperlipemia

### Prevalence of CKD

The prevalence of CKD, weighted and estimated using IPW, is shown in Table 2. CKD, defined as eGFR < 60 or positive proteinuria, accounted for 25.3% of all participants. CKD stage G3aA1 (eGFR is 45 to less than 60 and negative proteinuria) represented 16.6% of the total, belonging to the category of mild renal dysfunction with negative proteinuria. To further examine the difference of age on the prevalence of CKD, we estimated the prevalence of CKD in the age groups 65–75 years and 75 years and older and found that the prevalence was higher in the older age groups: approximately 11.8% in the age group 65–75 years and 34.6% in the age group 75 years and older (Table 3). Additionally, we explored the prevalence of comorbidities considered related to CKD among those who underwent health screening. Hypertension was observed in 66.8% of patients with CKD compared with 54.8% of those without CKD, and diabetes was observed in 18.2% of the patients with CKD and 13.9% of those without CKD. Moreover, BMI levels were also assessed: 31.8% of patients with CKD and 23.0% of those without CKD had a BMI of 25 or higher (Table 4).

**Table 2:** Prevalence of CKD expressed in heatmap

Prevalence (%) [95% CI]	A1	A2	A3	All
G1	0.039 [0.034–0.044]	0.0029 [0.0018–0.0045]	< 0.001	0.042 [0.037–0.047]
G2	74.68 [74.5–74.85]	2.33 [2.27–2.39]	0.092 [0.080–0.10]	77.09 [76.92–77.27]
G3a	16.61 [16.46–16.76]	1.01 [0.97–1.05]	0.061 [0.052–0.072]	17.68 [17.52–17.84]
G3b	3.73 [3.64–3.81]	0.64 [0.60–0.67]	0.080 [0.068–0.092]	4.44 [4.35–4.54]
G4	0.44 [0.41–0.47]	0.21 [0.19–0.23]	0.042 [0.034–0.051]	0.69 [0.65–0.73]
G5	0.014 [0.010–0.020]	0.037 [0.030–0.046]	0.0052 [0.0030–0.0092]	0.057 [0.048–0.068]
All	95.5 [95.41–95.58]	4.22 [4.14–4.31]	0.28 [0.26–0.30]	100

**Table 3:** Prevalence of CKD expressed in heatmap by age group 65–75 years

Prevalence (%) [95% CI]	A1	A2	A3	All
G1	0.095 [0.084–0.11]	0.0070 [0.0045–0.011]	< 0.001	0.10 [0.091–0.12]
G2	88.14 [88.01–88.27]	1.71 [1.66–1.76]	0.061 [0.052–0.071]	89.91 [89.79–90.02]
G3a	8.19 [8.09–8.30]	0.45 [0.42–0.48]	0.028 [0.022–0.035]	8.67 [8.56–8.78]
G3b	0.86 [0.83–0.90]	0.22 [0.20–0.24]	0.036 [0.029–0.044]	1.12 [1.08–1.16]
G4	0.072 [0.062–0.084]	0.075 [0.065–0.086]	0.018 [0.013–0.024]	0.16 [0.15–0.18]
G5	0.0078 [0.005–0.012]	0.026 [0.020–0.033]	0.0019 [0.00067–0.0055]	0.036 [0.029–0.044]
All	97.37 [97.31–97.43]	2.49 [2.43–2.55]	0.14 [0.13–0.16]	100

Prevalence (%) [95% CI]	A1	A2	A3	All
G1	–	–	–	–
G2	65.38 [65.1–65.66]	2.75 [2.66–2.85]	0.11 [0.095–0.13]	68.25 [67.97–68.52]
G3a	22.42 [22.17–22.66]	1.40 [1.33–1.47]	0.084 [0.069–0.10]	23.9 [23.65–24.15]
G3b	5.70 [5.56–5.85]	0.92 [0.87–0.98]	0.11 [0.092–0.13]	6.73 [6.58–6.89]
G4	0.69 [0.64–0.74]	0.30 [0.27–0.34]	0.058 [0.045–0.075]	1.05 [0.99–1.11]
G5	0.019 [0.012–0.029]	0.045 [0.034–0.060]	0.0075 [0.004–0.014]	0.071 [0.057–0.089]
All	94.21 [94.07–94.34]	5.42 [5.29–5.56]	0.37 [0.34–0.41]	100

**Table 4:** Prevalence of risk factor

Prevalence (%)	CKD	Non-CKD	*p* value
HT			
High blood pressure	33.7	29.7	
Medication	51.1	38.4	
Either	66.8	54.8	< 0.01
DM			
High blood glucose	13.5	10.1	
Medication	12.7	8.49	
Either	18.2	13.9	< 0.01
BMI			
< 18.5 kg/m^2^	4.6	7.3	
18.5–25.0 kg/m^2^	63.6	69.7	
≥ 25 kg/m^2^	31.8	23.0	< 0.01

*HT* Hypertension, *DM* Diabetes mellitus, *HL* Hyperlipemia

High Blood Pressure: Systolic blood pressure ≥ 140 mmHg or Diastolic blood pressure ≥ 90 mmHg

High Blood Glucose: HbA1c ≥ 6.5%

High LDL-C: LDL-C ≥ 140 mg/dL

**Fig. 1:**
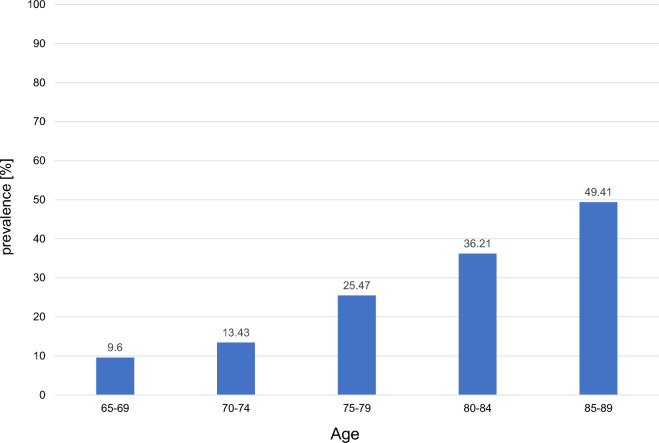
Prevalence of people diagnosed with CKD by age

We also calculated the prevalence of CKD among those who underwent health screenings. Patients with CKD accounted for 16.4%, and among them, those with G3aA1 accounted for 11.0%, which was lower than the value estimated using the IPW method (Online Resource Table 1). Furthermore, the prevalence of CKD was calculated for each age group in 5-year increments as follows: 9.6% for those aged 65–70 years, 13.43% for those aged 70–75 years, 25.47% for those aged 75–80 years, 36.21% for those aged 80–85 years, and 49.41% for those aged 85–90 years, showing an increase with age (Fig. 1).

### Model validation

The area under the curve (AUC) value for the estimated probability of not undergoing health screening in this study was 0.82, indicating gthe ood predictive performance of this prediction model (Online Resource Fig. 2). In addition, the calibration plots showed a good overall agreement, suggesting that the model accurately reflected the observed data (Online Resource Fig. 3).

## Discussion

In this study, we estimated the prevalence of CKD among the Japanese older population using health screening data. Among the older population aged 65–90 years, 25.3% were estimated to have CKD. The prevalence of CKD was even higher (34.6%) among the super-older population (those aged 75 years or older). Regarding comorbidities, hypertension and diabetes mellitus were tabulated, both of which were more common among those with CKD.

Prevalence estimates in Japan were reported to be approximately 13% in previous studies conducted 15 years ago, which examined the prevalence of CKD among participants in a nationwide annual health screening program in 11 prefectures in Japan [7]. Although the results of this study are widely recognized and important, the applicability of the study to a generalized population remains a limitation because it was conducted with a focus on health screening participants. By analyzing only health screening participants, there is a possibility of selection bias in the more health-conscious population, and consideration must be given to the possibility of underestimating the prevalence of CKD. To overcome this issue, our study used the IPW method to estimate the prevalence of CKD, which showed a higher prevalence. The impact of non-health screening participants was significant, and the importance of considering these individuals was evident. This is the first study to estimate prevalence considering the entire population, and can be a highly suggestive study.

CKD is a significant health issue worldwide, and its prevalence has been reported in other countries. According to a previous study using data from the NHANES surveys in the United States, the prevalence of stage 3 and 4 CKD was estimated to be 6.9%, showing an increasing trend with age. The prevalence of CKD in the late 1990s and the early 2000s was on the rise, but since 2003–2004, the prevalence rate has been reported to have stabilized [14]. Moreover, the prevalence rates and mortality risks in Asian countries have been reported [15–17]. Although general comparisons are difficult owing to the differences in creatinine measurement methods and the definition of urinary proteins in different countries, the prevalence of CKD is not low. In this study, the prevalence of CKD among the older population in Japan was higher than that reported in other countries. Given the advancing aging population in Japan, a higher prevalence compared to other countries is considered a relatively reasonable outcome. The prevalence of CKD is expected to increase owing to the global aging of society. Estimating the prevalence of CKD, especially in Japan, where the population is aging, is useful for solving mid- to long-term public health issues and may prevent CKD progression.

This study also estimated the prevalence of each stage of CKD, finding that 17.7% of the patients were classified as G3a, 4.4% as G3b, and 0.8% as G4 or later. Among those over 75 years of age, the prevalence of G3a was 23.9%. We identified a significant number of older patients with mild renal dysfunction and negative proteinuria, particularly in the G3aA1 stage. This pattern was notably pronounced in the population over 75 years of age. This suggests the need to consider the physiological decline in renal function caused by aging. It is well-documented that renal function declines with age [18]. Therefore, renal function decline is not always clinically significant. There has been previous discussion about the definition of CKD in the older population [19]. Uniform management is not appropriate because many older patients have decreased eGFR, and age is a major modifier [20, 21]. In addition, some studies have proposed adding age-specific thresholds for GFR to the definition of CKD, because the GFR threshold at which the risk of death increases is not consistent across all ages [22]. However, a previous study stated that older individuals with mild renal dysfunction are not necessarily at a lower risk of CKD complications, in contrast to the variability of criteria with age [23]. Thus, the criteria for diagnosing CKD in the older population have been investigated. Our study revealed a high prevalence of CKD in the older population, but its clinical significance needs to be examined.

Our study also included a large population of patients with CKD and mildly impaired renal function. However, we were not able to assess the outcomes of patients with CKD at each stage. That is, we were unable to assess the incidence of cardiovascular disease, prognosis, or progression to end-stage renal failure in the patients. It is difficult to distinguish between age-related physiologic and pathological renal function impairment with clinical significance, especially in a population with mild renal dysfunction and negative proteinuria. Our study also indicated a high prevalence of hypertension in patients with CKD. A previous study suggested that hypertension in older patients with early-stage CKD may not necessarily lead to an apparent worsening of clinical outcomes [24], supporting the possibility that not all individuals in this CKD population require medical intervention. In the early stage of CKD, a controversial area among the older population, there is a need for additional studies, including the evaluation of outcomes to examine clinical meanings and identify populations in need of medical intervention. We plan to conduct further studies on this topic.

This study has several limitations. First, we conducted a cohort study using an insurance database. Although the database includes a large number of older people, among the older population, patients with end-stage renal failure—who are receiving hospital care or undergoing dialysis—are likely not to undergo health screenings. This leads to selection bias, especially in estimating the prevalence of patients with advanced CKD, which is likely to be underestimated even with weighted assessment. Indeed, the number of patients with CKD classified as G4 or G5 in this study was small, less than 1%, while the reported number of patients on chronic dialysis patients at the end of 2022 was approximately 340,000 individuals [2]. This discrepancy is one of the material limitations of this study. However, we believe that this study is useful for identifying patients with relatively early CKD in the older population and has clinical value. Second, this study was conducted using a database of health examinations based on data from annual or semiannual follow-up surveys. While the KDIGO guidelines [25] require multiple renal function measurements confirmed over at least 90 days to diagnose CKD, this study used the results of renal function measurements and urinalysis from two physical examinations to diagnose CKD. The interval between each eGFR measurement may be potentially long. Although many epidemiological studies often define CKD based on a single kidney function measurement, our study may have improved diagnostic accuracy by employing the results of two physical examinations. Third, this study did not examine the presence or absence of medical interventions for CKD, such as medications, in patients at each stage. The status of the intervention at each stage, as well as clinical outcome evaluations, need to be examined in the future. Additionally, we were unable to obtain the number of insurers. This database does not contain information on the number of insurers, including municipalities and companies, due to privacy concerns. We are considering future research using other database to address this issue.

In conclusion, the weighted prevalence of CKD among the older Japanese population was estimated to be approximately 25%. This prevalence increases with age, and most patients have mild renal function decline and negative proteinuria. In an aging society, the findings of this study are important for understanding CKD in older populations, and for developing appropriate medical interventions.

## Supplementary Information

Below is the link to the electronic supplementary material.

Supplementary file1 (PDF 112 KB)

Supplementary file2 (PDF 89 KB)

## Data Availability

No data are available. The data underlying this article are not shared because of the privacy policies of data providers.
