# Supplementary material for: Estimating the prevalence of chronic kidney disease in the older population using health screening data in Japan
Source: Clin Exp Nephrol. 2024 Oct 5;29(3):276–82. doi: 10.1007/s10157-024-02570-y (PMC11893708; doi:10.1007/s10157-024-02570-y)

Online Resource Figure 1. Diagram of participant selection

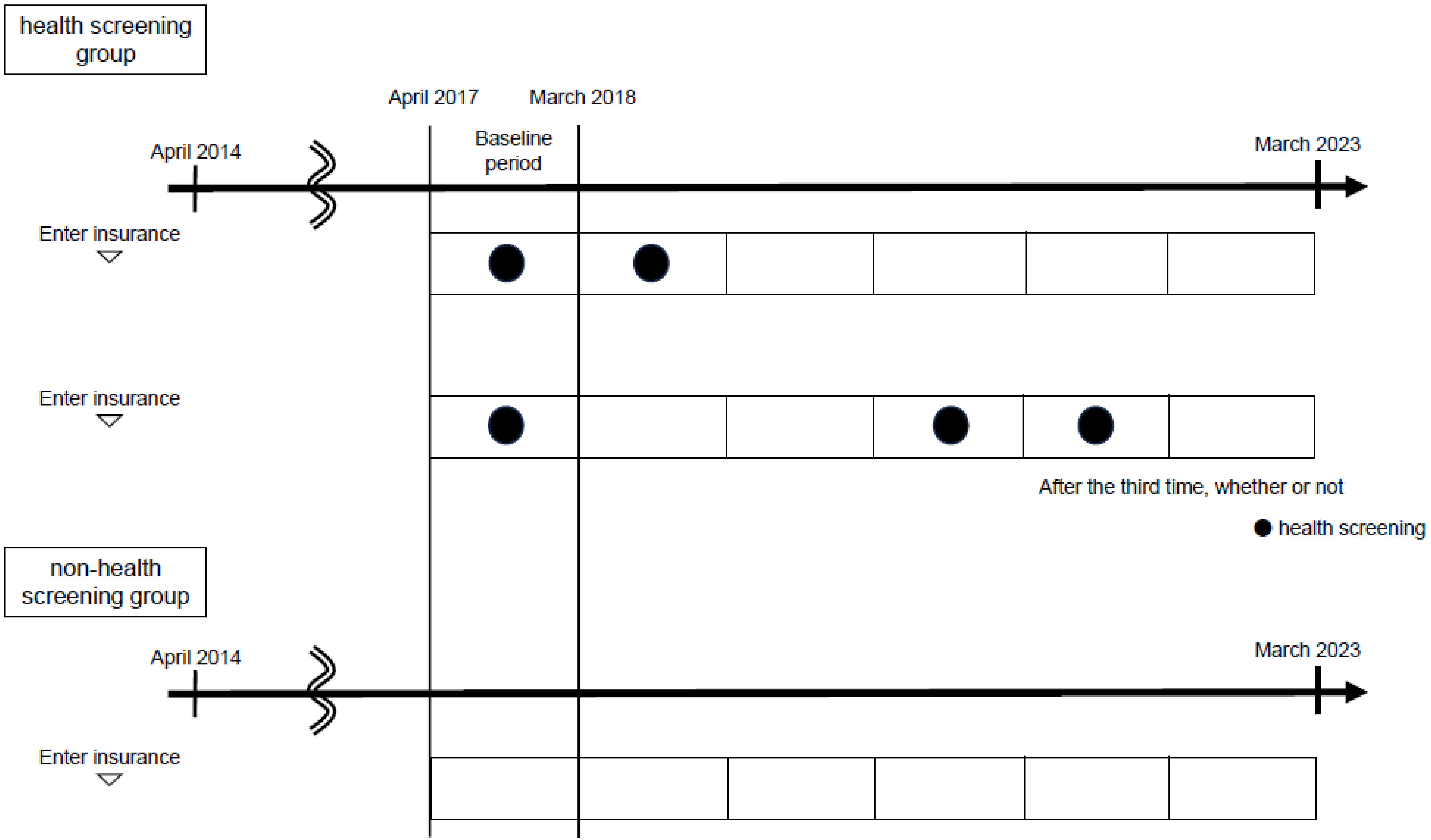

Online Resource Figure 2. ROC curve of this study's prediction model

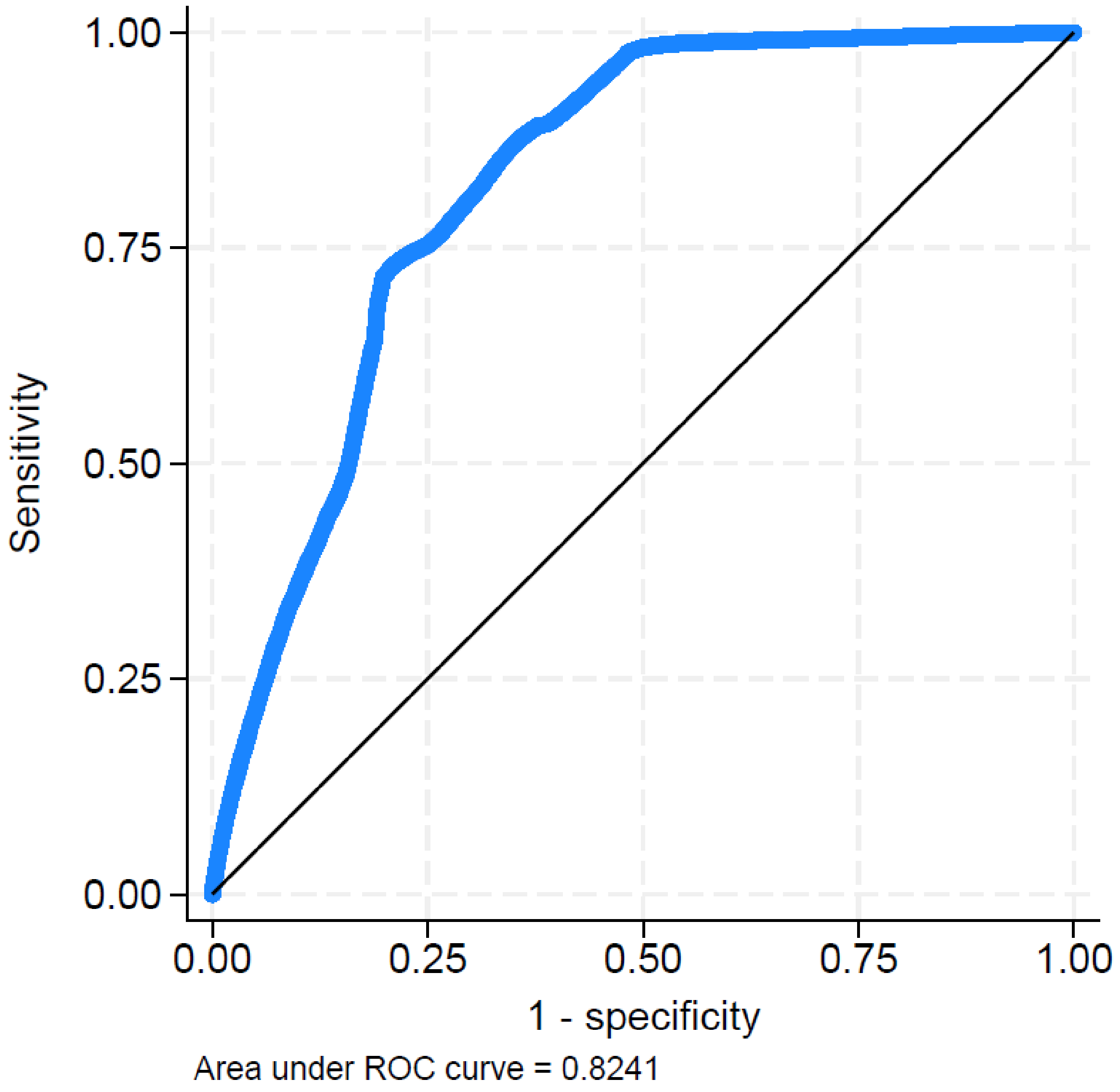

Online Resource Figure 3. Calibration plots between predicted probabilities and actual outcomes

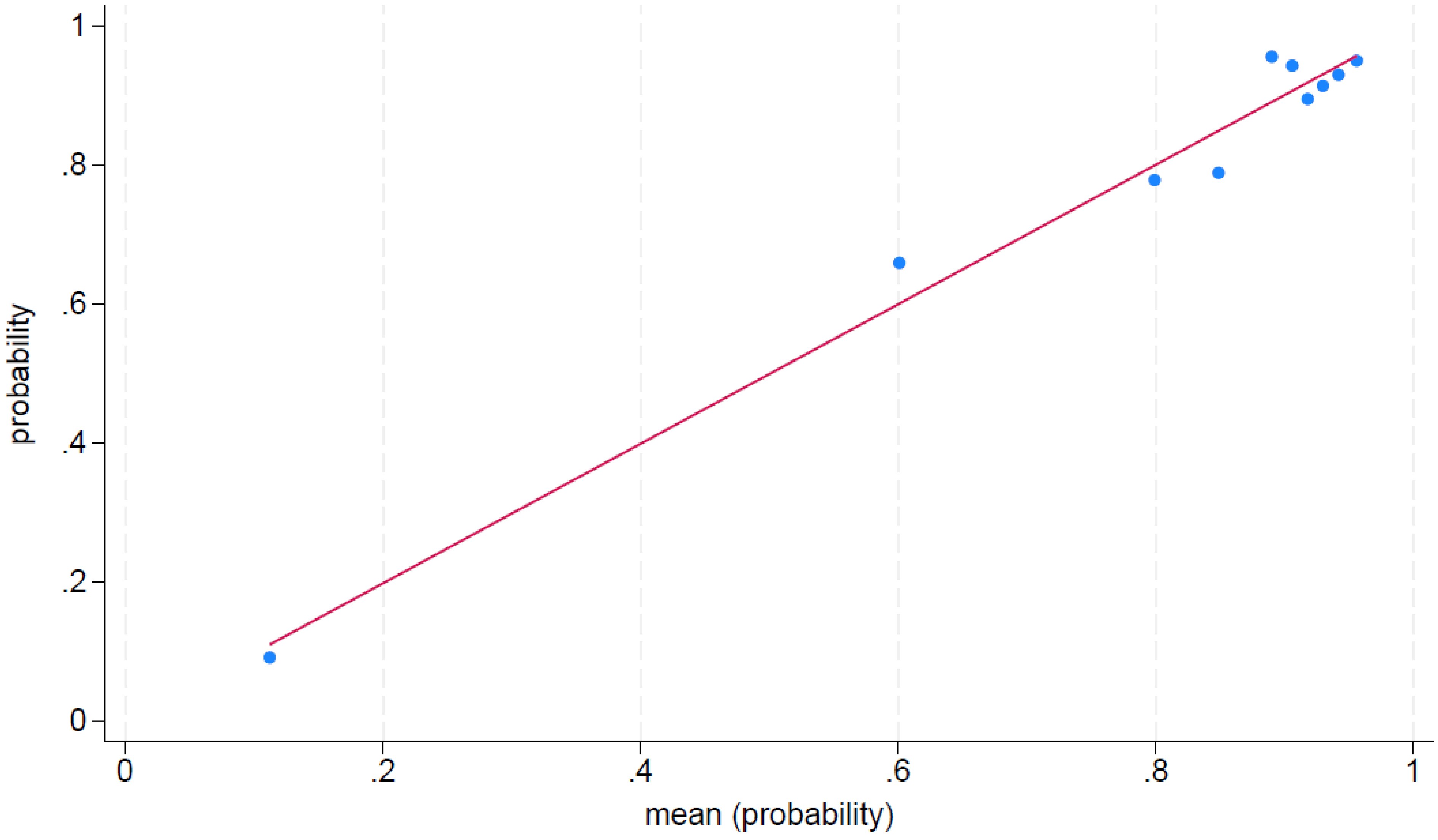

Supplement: Supplementary file 1 — Supplementary file1 (PDF 112 KB) [file 10157_2024_2570_MOESM1_ESM.pdf]
