# Supplementary material for: Estimating the prevalence of chronic kidney disease in the older population using health screening data in Japan
Source: Clin Exp Nephrol. 2024 Oct 5;29(3):276–82. doi: 10.1007/s10157-024-02570-y (PMC11893708; doi:10.1007/s10157-024-02570-y)
Supplement: Supplementary file 2 — Supplementary file2 (PDF 89 KB) [file 10157_2024_2570_MOESM2_ESM.pdf]

Online Resource_Table 1 Prevalence of CKD in heatmap using health screening data

Prevalence (%)	A1	A2	A3	All
G1	0.07	0.01	—	0.08
G2	83.58	1.93	0.07	85.58
G3a	10.97	0.65	0.04	11.66
G3b	1.87	0.38	0.05	2.30
G4	0.21	0.13	0.03	0.37
G5	0.01	0.03	—	0.04
All	96.71	3.13	0.19	100
